# Transcription Factor GmWRKY142 Confers Cadmium Resistance by Up-Regulating the Cadmium Tolerance 1-Like Genes

**DOI:** 10.3389/fpls.2020.00724

**Published:** 2020-06-03

**Authors:** Zhandong Cai, Peiqi Xian, Huan Wang, Rongbin Lin, Tengxiang Lian, Yanbo Cheng, Qibin Ma, Hai Nian

**Affiliations:** ^1^The State Key Laboratory for Conservation and Utilization of Subtropical Agro-bioresources, South China Agricultural University, Guangzhou, China; ^2^The Key Laboratory of Plant Molecular Breeding of Guangdong Province, College of Agriculture, South China Agricultural University, Guangzhou, China; ^3^Guangdong Laboratory for Lingnan Modern Agriculture, Guangzhou, China; ^4^The Guangdong Subcenter of the National Center for Soybean Improvement, College of Agriculture, South China Agricultural University, Guangzhou, China

**Keywords:** transcription factor, WRKY, cadmium stress, CDT1-likes, soybean

## Abstract

Cadmium (Cd) is a widespread pollutant that is toxic to living organisms. Previous studies have identified certain WRKY transcription factors, which confer Cd tolerance in different plant species. In the present study, we have identified 29 Cd-responsive *WRKY* genes in Soybean [*Glycine max* (L.) Merr.], and confirmed that 26 of those *GmWRKY* genes were up-regulated, while 3 were down-regulated. We have also cloned the novel, positively regulated *GmWRKY142* gene from soybean and investigated its regulatory mechanism in Cd tolerance. GmWRKY142 was highly expressed in the root, drastically up-regulated by Cd, localized in the nucleus, and displayed transcriptional activity. The overexpression of *GmWRKY142* in *Arabidopsis thaliana* and soybean hairy roots significantly enhanced Cd tolerance and lead to extensive transcriptional reprogramming of stress-responsive genes. *ATCDT1*, *GmCDT1-1*, and *GmCDT1-2* encoding cadmium tolerance 1 were induced in overexpression lines. Further analysis showed that *GmWRKY142* activated the transcription of *ATCDT1*, *GmCDT1-1*, and *GmCDT1-2* by directly binding to the W-box element in their promoters. In addition, the functions of *GmCDT1-1* and *GmCDT1-2*, responsible for decreasing Cd uptake, were validated by heterologous expression in *A. thaliana*. Our combined results have determined GmWRKYs to be newly discovered participants in response to Cd stress, and have confirmed that *GmWRKY142* directly targets *ATCDT1*, *GmCDT1-1*, and *GmCDT1-2* to decrease Cd uptake and positively regulate Cd tolerance. The GmWRKY142-GmCDT1-1/2 cascade module provides a potential strategy to lower Cd accumulation in soybean.

## Introduction

Cadmium (Cd) is a non-essential metallic trace element that is toxic to both plants and animals. Recently, global climate change and rapid industrialization have contributed to an increase in Cd deposition in soil, leading to a major global threat to crop productivity and human health (Das et al., 1997; Moulis and Thévenod, 2010; Clemens et al., 2013; Okereafor et al., 2020; Zheng et al., 2020). The cultivation of cadmium tolerant crops and reduction of cadmium concentration in the edible parts of plants are solutions that could potentially alleviate the risks to human health (Grant et al., 1998; Gill and Tuteja, 2011; Tang et al., 2017; Zhao and Huang, 2018). To this effect, it is necessary to understand the mechanisms of Cd tolerance in plants and to investigate important genes encoding Cd tolerance.

Plants have evolved a set of versatile adaptive mechanisms to cope with Cd stress. These mainly involve the use of enzymatic and non-enzymatic antioxidants, the extrusion of Cd across the plasma membrane, the restriction of Cd movement to roots by mycorrhizas, and finally, the sequestration of metals in metabolically inactive parts such as root cell walls and leaf vacuoles (Dong et al., 2007; Hasan et al., 2009; Nagajyoti et al., 2010; Gallego et al., 2012; Rizwan et al., 2017; Shanying et al., 2017; Kumar et al., 2019; Sheng et al., 2019; Zhang Z. H. et al., 2019). Considerable progress has recently been made in understanding the molecular mechanisms of Cd accumulation in plants. Several key genes encoding metal transporters, chelator proteins, antioxidant enzymes, defensin genes and transcription factors have been reported to participate in Cd detoxification and tolerance in plants (Zhu et al., 1999a, b; Wu et al., 2012; Sasaki et al., 2014; Chen et al., 2016; Yang et al., 2016; Cai et al., 2017; Luo et al., 2019a, b; Mekawy et al., 2020). Of these functional proteins, Cysteine (Cys) -rich proteins are considered the most important Cd chelator proteins and generally have relatively low molecular weights (4–14 kDa) with a high ratio of cysteine residues (Song et al., 2004; Kuramata et al., 2009). Since the characterization of the first Cd-binding Cys-rich membrane protein from horse kidneys in 1957 (Margoshes and Vallee, 1957), a number of Cd-binding Cys-rich genes have been identified in plants, including *AtPcr1* (Song et al., 2004), *ThMT3* (Yang et al., 2011), *OsDEP1* (Kunihiro et al., 2013), *CnMT1* and *CnMT2* (Palacios et al., 2014), and *OsMT-3a* (Mekawy et al., 2020). Particularly, DcCDT1 (*Digitaria ciliaris* cadmium tolerance 1) and its homologues (AtCDT1 and OsCDT1) are specific to higher plants; they are unique and distinctive in both their peptide lengths (49–60 amino acids) and in their arrangement of Cys residues in the consensus sequence of CL-(Y/F)-A-(C/T)-X5-CC-(F/C)-CCYE-(T/K)-C-(E/K)-C-(CLDCL or delete)-CCCC (Kuramata et al., 2009; Matsuda et al., 2009). Transgenic *A. thaliana* plants or yeast cells overexpressing *DcCDT1*, *OsCDT1*, or *AtCDT1* consistently displayed a Cd tolerant phenotype and accumulated lower amounts of Cd. Moreover, 5 *DcCDT1* homologs in rice (OsCDT1 – 5) were up-regulated to varying degrees by Cd treatment (Kuramata et al., 2009). However, the mechanism of CDT1 regulation by Cd stress remains to be elucidated.

Transcription factors (TFs) are potentially the core components in the regulatory networks of Cd detoxification and tolerance owing to their functions as key regulators of the Cd stress response via their control on downstream gene expression. Most types of transcription factors regulating Cd detoxification and tolerance have been identified in plants, including metal response element transcription factors (MTF) (Smirnova et al., 2000; Solis et al., 2002; Lin et al., 2017), basic helix-loop-helix (bHLH) transcription factors (Wu et al., 2012; Yao et al., 2018), myeloblastosis (MYB) transcription factors (Hu et al., 2017; Xu et al., 2018; Zhang P. et al., 2019), ethylene responsive factors (ERF) (Yi et al., 2004; Tang et al., 2005; Lin et al., 2017), SQUAMOSA PROMOTER-BINDING PROTEIN-LIKE (SPL) transcription factors (Chen et al., 2018), Zn-regulated (Zip) transporters (Liu et al., 2019; Wu et al., 2019), and WRKY transcription factors (Yang et al., 2016; Hong et al., 2017; Han et al., 2019; Sheng et al., 2019). The WRKY proteins are a superfamily of transcription factors with up to 100, 90, and 170 representatives in Arabidopsis, Rice (*Oryza sativa* L.) and Soybean (*Glycine max*. L), respectively (Eulgem et al., 2000; Song et al., 2016; Xu et al., 2016; Yang et al., 2017). The first gene encoding WRKY transcription factor in plants was identified more than 20 years ago (Ishiguro and Nakamura, 1994; Rushton et al., 1995, 1996), and substantial progress in this area of research has since been achieved. Generally, WRKY proteins, composed of a WRKY domain (WRKYGQK) and a novel zinc-finger-like motif (Cx4–5Cx22–23HxH or Cx7Cx23HxC) at the N-terminal, can specifically recognize and bind the cis-acting W-box elements (TTGACC/T) of downstream genes (Eulgem et al., 2000; Eulgem and Somssich, 2007; Rushton et al., 2010). Several studies have found that WRKY TFs participate in the response to Cd stress by regulating the expression of downstream target genes such as *AtWRKY12* (Han et al., 2019), *AtWRKY13* (Sheng et al., 2019), *ThWRKY7* (Yang et al., 2016), and *CaWRKY41* (Dang et al., 2019). However, little is known about the role of the soybean WRKY TFs in Cd tolerance.

Soybean is important oil crops and the plant proteins resources widely grown around the world. Heavy metal contamination is an important factor that seriously inhibits soybean growth and threatens the human health. A better understanding of how soybean responds to heavy metal contamination would lay the foundations for developing effective countermeasures. Using the comprehensive mRNA transcriptome of soybean under Cd stress, 29 Cd-responsive WRKY genes were identified and confirmed by qRT-PCR analyses. Of these, *GmWRKY142* was selected to verify the function of Cd tolerance, due to its higher expression in root under normal conditions, as well as its strong up-regulation under Cd stress. We demonstrated that *GmWRKY142* overexpression in *A. thaliana* and soybean hair roots resulted in increased Cd tolerance and decreased Cd accumulation. Further analysis indicated that *GmWRKY142* directly activated *ATCDT1*, *GmCDT1-1*, and *GmCDT1-2* expression to enhance Cd tolerance. In summary, the GmWRKY142-GmCDT1-1/2 cascade module is potentially useful for the production of soybean with tolerance to Cd stress along with decreased Cd accumulation in their edible parts.

## Materials and Methods

### Plant Materials and Growth Conditions

The soybean cultivar “Huaxia 7” was used to perform RNA sequencing (RNA-seq) analyses, mRNA expression and phenotype profiling. Plump seeds of a similar size were surface sterilized in 75% alcohol followed by germination for 3 days in sterile vermiculite. Seedlings of a similar size were transplanted to modified 1/2 Hoagland solution (pH 5.8) containing the following macronutrients KNO_3_ (2.5 mM), Ca(NO_3_)_2_⋅4H_2_O (2.5 mM), MgSO_4_⋅7H_2_O (3.5 mM), and KH_2_PO_4_ (0.5 mM), and the micronutrients Fe-EDTA (50 μM), H_3_BO_3_ (7.5 μM), (NH_4_)_6_Mo_7_O_24_ (2.5 μM), MnCl_2_ (1.25 μM), ZnSO_4_ (1 μM), and CuSO_4_ (0.5 μM) (Hoagland and Arnon, 1950; Dang et al., 2019) with or without Cd, in an illuminated growth incubator (Model GXZ-300D; Ningbo, China) under a 16 h light/8 h dark photoperiod, for subsequent RNA-seq, gene cloning, and transcriptional expression experiments. For the Cd dose-response experiment, seedlings were subjected to modified 1/2 Hoagland solution containing 0, 5, 10, 15, 25, or 50 μM of CdCl_2_ for 2 h. During the time-course experiment, seedlings were subjected to modified 1/2 Hoagland solution with 25 μM CdCl_2_ for 0, 1, 2, 4, 6, 8, 12, or 24 h. Seedlings cultured in modified 1/2 Hoagland solution with 0 or 25 μM of CdCl_2_ for 4 h were sampled and subjected to RNA-seq analysis (LC-Bio, Hangzhou, China), according to the vendor’s protocol. All the experiments were performed with three independent biological replicates.

All *A. thaliana* seeds including the wild-type (WT) ecotype Col-0, and transgenic plants were surface-sterilized and transferred to sterilized matrix soil (Jiffy, Oslo, Norway). Following vernalization in the dark at 4°C for 3 days, the seeds were cultivated in an illuminated growth incubator (Model GXZ-300D, Ningbo, China) under a 16 h light / 8 h dark photoperiod at 24°C.

### RNA-Seq and Bioinformatics Analysis

RNA-seq analysis was performed by LC-Bio company (Hangzhou, China). For the transcriptomic analysis of Cd stress in soybean, 3-day old seedlings were cultured in modified 1/2 Hoagland solution with 0 or 25 μM of CdCl_2_ for 4 h were sampled and subjected to RNA-seq analysis. For the *Arabidopsis thaliana*, 2-week old seedlings of both the WT (Col-0) and the GmWRKY142-OX line (Line 6) under non-stress conditions were used for transcriptomic analysis. Following the manufacturer’s procedure, total RNA was extracted from the samples using the Spectrum Plant Total RNA Kit (Sigma-Aldrich, St. Louis, MO, United States, STRN10-1KT) and mRNA was enriched and fragmented into shorter fragments by mixed with fragmentation buffer. First-strand cDNA was synthesized from fragmented mRNA using random hexamer primer. End repair of the double-stranded cDNAs was performed using T4 polynucleotide kinase, T4 DNA polymerase and DNA polymerase I Klenow fragment. Then, T4 DNA ligase was used to ligate the fragments to adapters. The available fragments were selected and then enriched by PCR amplification. The constructed libraries were qualified and quantified using an Agilent 2100 Bioanaylzer and the ABI StepOnePlus Real-Time PCR System and then sequenced via Illumina Novaseq^TM^ 6000. The obtained raw reads were then cleaned by removing the low-quality reads and/or adaptor sequences. The clean reads were mapped to reference sequences using SOAPaligner/SOAP2 (Li et al., 2009). The reference genomes and genes set of Soybean and Arabidopsis thaliana were downloaded from the NCBI (National Center for Biotechnology Information) site^1^^,^^2^. The gene expression levels were calculated using the reads per kilobase per million reads method according to (Mortazavi et al., 2008). Subsequently, based on sequence homology, the differentially expressed genes by gene ontology terms^3^ were imported into Blast2GO, a software package that retrieves GO terms, allowing gene functions to be determined and compared. To further understand the biological pathways in which the differentially expressed genes are involved, the differentially expressed genes were compared against the KEGG database^4^. Based on reports of the soybean WRKY gene family from previous studies and differentially expressed genes annotation in this study, probable WRKY transcription factors in soybean were identified and named according to (Yu et al., 2016) (Supplementary Table S3). The sequences for both the soybean and *Arabidopsis thaliana* used in the present study can be downloaded from the Phytozome database (Version 12)^5^ according the gene numbers.

### RNA Isolation and qRT-PCR Analysis

Total RNA was isolated from samples using the Plant Total RNA Purification Kit (TR02-150, GeneMarkbio, Taiwan, China). The first-strand cDNA was reverse transcribed by PrimeScript^TM^ RT reagent Kit with a gDNA Eraser kit (RR047, Takara Bio, Shiga, Japan). qRT-PCR analysis was carried out using TB Green^TM^ Premix Ex Taq^TM^ II (RR820, Takara Bio) and CFX96 Real-Time PCR Detection System (Bio-Rad, Hercules, CA, United States). In all experiments, qRT-PCR analyses were performed as triplicates on three different RNA samples isolated independently from each tested condition. Relative gene expression levels were detected using the 2^–△^^△^^Ct^ algorithm (Livak and Schmittgen, 2001) normalized to the expression of the Actin genes *GmACT3* (GenBank: AK285830.1) or *AtActin2* (GenBank: AT3g18780). Three independent biological replicates were tested in each experiment for the qRT-PCR analysis. Primers used are listed in Supplementary Table S1.

### DNA and cDNA Clones, Vector Construction, and Plant Transformation

The cDNA sequences of *GmWRKY142*, *GmCDT1-1* and *GmCDT1-2* genes were isolated using specific primers (Supplementary Table S1) and inserted into the *Xba* I and *Sac* I sites of the pTF101.1 vector, using the ClonExpress^®^ II One Step Cloning Kit (C112, Vazyme, Nanjing, China). The resulting plasmid was mobilized into *Agrobacterium* strains GV3101 and K599 by heat shock, and subsequently used to transform Arabidopsis and soybean hairy roots according to the *Agrobacterium*-mediated floral dip (Clough and Bent, 1998) and *Agrobacterium rhizogenes*-based transformation (Matthews and Youssef, 2016) methods, respectively. The complete coding sequence of *GmWRKY142* was cloned into the pCambia 1302, pGBKT7, pGADT7, and pGreenII 62-SK binary vectors using the ClonExpress^®^ II One Step Cloning Kit (C112, Vazyme) for the determination of subcellular localization, transcriptional activation activity assay, Yeast one-hybrid assay and Dual LUC assay, respectively. The genomic DNA of Huaxia 7 and WT plants were extracted using the Plant Genomic DNA Kit (DP305, TIANGEN, Beijing, China). Upstream sequences spanning 2,000 bp from the start codons of the *ATCDT1*, *GmCDT1-1*, and *GmCDT1-2* genes were analyzed to determine the W-box elements. The original *ATCDT1*, *GmCDT1-1*, and *GmCDT1-2* promoter fragments containing a genuine W-box element were amplified by PCR, while mutated W-box elements were identified by a change in the W-box sequence into “TAAAAT.” Either original or mutated fragments were cloned into the pAbAi vector using the ClonExpress^®^ II One Step Cloning Kit (C112, Vazyme) and used as baits in yeast one-hybrid assays. Details of primers used in vector construction are listed in Supplementary Table S1.

### Subcellular Localization

Subcellular localization was investigated by transiently overexpressing *35S:GmWRKY142-GFP* in tobacco (*Nicotiana tabacum*) leaves via *Agrobacterium*-mediated transformation (Sparkes et al., 2006). Nuclear dye (DAPI) and GFP fluorescence signals were observed using confocal scanning microscopy (Model LSM780, Zeiss, Jena, Germany).

### Yeast One-Hybrid Assay

This assay was performed using the MATCHMAKER^®^ Gold Yeast One-Hybrid Library Screening System (Clontech) and the YEASTMAKER^TM^ Yeast Transformation System 2 (Clontech). The full-length coding sequence of *GmWRKY142* was cloned from cDNA and inserted into the effector construct pGADT7. A DNA fragments that consisted of two W-box motifs (TATGCTTTA GCTGGAATTGACTTCACCAGGTTTGACCTTACAGGTAGG TAGTTGAGT) or their mutants (ATATGCTTTAGCTGGAA TAAAATTCACCAGGTTAAAACTTACAGGTAGGTAGTTGA GT) were synthesized and introduced into the upstream region of the mini-promoter of AurR (pAbAi), which were termed pAbAi-Wbox and pAbAi-mWbox, respectively. The promoter sequences of *GmCDT1-1*, *GmCDT1-2*, and *AtCDT1* were amplified and introduced into the upstream region of the mini-promoter of AurR, respectively. Prey and reporter vectors were co-transformed into the yeast strain Y1H Gold. Cells were grown in SD/-Leu liquid media to an OD_600_ of 0.1 and diluted 10-fold with normal saline. For each dilution, a volume of 7 μL was spotted on SD/-Leu media plates containing either 0 or 150 ng mL^–1^ AbA in order to test the strength of the interaction. Plates were incubated for 3–4 days at 30°C.

### Cd Tolerance Assay

Twenty-five-day-old plants grown in pots (length × width × height = 7.0 × 7.0 × 7.6 cm) were placed in trays and saturated either with 1/10 Hoagland solution (as control) or with 1/10 Hoagland solution containing 500 μM of CdCl_2_ solution for 10 days. Pots were then transferred to trays containing normal culture medium to promote plant recovery for 12–15 days, after which the plants’ fresh weight and height were measured. Cd content was also measured using an atomic absorption spectrometer (Model AA-6800, Shimadzu Corporation, Japan). The experiment was performed with three independent biological replicates.

### Statistical Analysis

All data were analyzed using GraphPad Prism^®^ 5 (Version 5.01, GraphPad Software, Inc., United States) for calculating mean and standard deviation. At least three biological replicates were included in the data, and all data were analyzed using ANOVA or Duncan’s test for the determination of the significant differences with SPSS 21 (IBM Corp, 2012).

## Results

### The GmWRKY Family of Genes Responds to Cd Stress in Soybean

To identify Cd-responsive WRKY family members in soybean, genome wide transcriptomic analysis was conducted with Huaxia 7 roots exposed to Cd stress. By comparing the transcriptome expression patterns between control and Cd treated groups, a total of 5,285 differentially expressed genes (DEGs) were identified in soybean plants under Cd stress (Supplementary Table S2). Based on reports from previous studies, the soybean genome contains at least 170 WRKY family members (Song et al., 2016; Yang et al., 2017). However, genome wide transcriptomic analysis revealed that only 29 genes were up- or down-regulated more than 1.5-fold in roots in response to Cd stress (Table 1). Of these, 26 GmWRKY genes in the roots were up-regulated, while 3 were down-regulated. Furthermore, qRT-PCR analyses of all 29 Cd-responsive WRKY genes confirmed their differential expression in response to Cd stress (Table 1). It should be noted that *GmWRKY142* registered a higher fragments per kilobase of transcript per million mapped reads (FPKM) under normal conditions, as well as being strongly up-regulated by Cd stress, which prompted our interest in further research.

**TABLE 1:** GmWRKY family genes respond to Cd stress.

**Gene ID of NCBI**	**Gene numbers**	**Gene name**	**Fragments Per kilobase per million (FPKM)**						**Fold change (FC)**	**Log2(FC)**	**qRT-PCR**
			**CK_1**	**CK_2**	**CK_3**	**Cd_1**	**Cd_2**	**Cd_3**			
100781603	*Glyma.15G186300*	*GmWRKY146*	3.89	3.23	2.47	1.13	0.68	0.69	0.26	–1.94	-4.33 ± 0.23
100788213	*Glyma.09G129100*	*GmWRKY95*	1.27	1.29	0.91	0.61	0.10	0.22	0.27	–1.90	-4.89 ± 0.52
100783661	*Glyma.19G221700*	*GmWRKY184*	10.66	6.40	10.14	3.12	2.35	3.11	0.32	–1.66	-3.52 ± 0.34
100127370	*Glyma.01G224800*	*GmWRKY7*	2.42	0.97	0.90	3.14	5.27	4.05	2.90	1.54	2.35 ± 0.84
100127423	*Glyma.03G220100*	*GmWRKY25*	2.65	1.32	3.06	8.96	7.07	4.43	2.91	1.54	2.58 ± 0.09
100802124	*Glyma.15G110300*	*GmWRKY142*	33.19	30.90	27.40	76.42	104.34	92.06	2.98	1.58	4.02 ± 0.52
100127367	*Glyma.01G128100*	*GmWRKY4*	2.18	2.36	0.82	5.55	6.03	4.63	3.02	1.60	2.36 ± 0.07
100127421	*Glyma.11G163300*	*GmWRKY114*	11.68	10.37	13.61	31.62	46.88	34.56	3.17	1.66	3.67 ± 0.10
100170682	*Glyma.18G213200*	*GmWRKY172*	0.12	0.48	1.41	2.66	1.44	2.53	3.30	1.72	2.48 ± 0.12
100797998	*Glyma.12G097100*	*GmWRKY115*	0.93	0.67	0.12	1.93	2.32	1.74	3.48	1.80	3.47 ± 0.25
100127387	*Glyma.03G256700*	*GmWRKY28*	1.32	0.41	0.61	2.45	3.76	2.27	3.61	1.85	3.41 ± 0.33
100807966	*Glyma.07G023300*	*GmWRKY68*	3.93	2.48	5.31	12.84	13.75	15.90	3.62	1.86	4.08 ± 0.41
732588	*Glyma.15G003300*	*GmWRKY141*	3.02	3.59	4.41	11.78	16.75	15.86	4.03	2.01	3.59 ± 0.53
100795177	*Glyma.06G307700*	*GmWRKY66*	1.85	2.13	1.23	7.68	8.35	7.12	4.44	2.15	4.13 ± 0.22
102666898	*Glyma.03G042700*	*GmWRKY21*	2.34	2.68	1.48	8.34	11.75	11.64	4.88	2.29	4.53 ± 0.84
100127375	*Glyma.08G021900*	*GmWRKY78*	0.58	0.85	0.58	2.10	4.09	4.09	5.13	2.36	5.55 ± 1.19
100787050	*Glyma.05G127600*	*GmWRKY44*	0.29	0.36	0.26	1.82	1.36	1.82	5.47	2.45	5.58 ± 0.98
100170747	*Glyma.16G026400*	*GmWRKY147*	0.87	0.77	0.70	5.04	7.62	4.47	7.31	2.87	8.22 ± 1.14
100798500	*Glyma.04G061400*	*GmWRKY31*	0.12	0.20	0.15	1.74	1.39	1.01	8.89	3.15	8.47 ± 1.21
100790050	*Glyma.19G254800*	*GmWRKY185*	0.17	0.45	0.74	4.05	4.74	4.00	9.41	3.23	10.77 ± 1.49
100779533	*Glyma.18G092200*	*GmWRKY168*	0.27	0.77	0.38	4.28	5.13	4.97	10.13	3.34	12.36 ± 1.37
100816891	*Glyma.08G082400*	*GmWRKY81*	0.32	0.65	0.28	4.53	5.10	5.47	12.11	3.60	14.56 ± 2.56
100812027	*Glyma.06G125600*	*GmWRKY56*	0.21	0.48	0.09	3.03	3.24	3.82	12.94	3.69	15.21 ± 1.74
100127378	*Glyma.08G011300*	*GmWRKY76*	0.06	0.27	0	0.31	2.27	1.73	13.30	3.73	13.97 ± 1.55
100127373	*Glyma.06G061900*	*GmWRKY53*	0.02	0.21	0.22	2.05	2.66	1.68	13.97	3.80	15.20 ± 2.48
100776906	*Glyma.04G238300*	*GmWRKY39*	0.05	0.06	0.03	0.31	0.67	1.38	18.34	4.20	20.24 ± 3.0
102667019	*Glyma.01G222300*	*GmWRKY6*	0.05	0.11	0	0.36	2.24	1.54	26.11	4.71	27.55 ± 2.76
100798375	*Glyma.13G370100*	*GmWRKY126*	0.03	0.07	0.28	2.26	4.43	4.22	28.21	4.82	28.42 ± 2.45
732586	*Glyma.19G094100*	*GmWRKY180*	1.04	0.48	0.64	20.04	30.36	28.53	36.42	5.19	44.87 ± 5.64

### Expression Patterns of *GmWRKY142*

**FIGURE 1:**
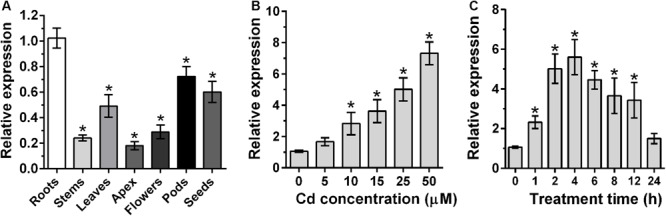
Analysis of expression patterns of GmWRKY142. **(A)** qRT-PCR analysis of the GmWRKY142 transcript in different tissues of the soybean variety Huaxia 7. mRNAs were isolated from roots, stems, leaves, apex, flowers, pods, and seeds. **(B)** Dose-dependent GmWRKY142 expression in roots. Roots were exposed to different Cd concentrations (0, 5, 10, 15, 25, and 50 μM) for 6 h. **(C)** For the time-course experiment, seedlings were exposed to 25 μM CdCl_2_ for 0, 1, 2, 4, 6, 8, 12, or 24 h. Samples were separately harvested for qRT-PCR analysis. Values are expressed as the means ± SD (*n* = 3). The experiment was performed with at least three independent biological replicates. Significant differences according to the one-way analysis of variance are denoted as follows: *p < 0.01.

To further characterize *GmWRKY142*, qRT-PCR was employed to analyze tissue samples and determine Cd-induced expression patterns. As shown in Figure 1A, *GmWRKY142* expression was detected in all examined tissues, with higher expression levels in roots, pods, seeds and leaves than stems, apex and flowers. To examine the expression patterns of *GmWRKY142* following Cd stress, 5-day-old soybean roots were exposed to different Cd concentrations for varying periods of time. After 2 h of treatment, *GmWRKY142* expression was significantly up-regulated by 10–50 μM Cd, whereby the fold change increased with increasing concentration (Figure 1B). Cd-induced *GmWRKY142* expression was also observed in a time-dependent manner, whereby treatment with 25 μM Cd resulted in the highest 5.6-fold expression to be reached at the 4th hour, followed by a decrease at the 6th hour (Figure 1C).

### Cloning and Characterization of *GmWRKY142*

**FIGURE 2:**
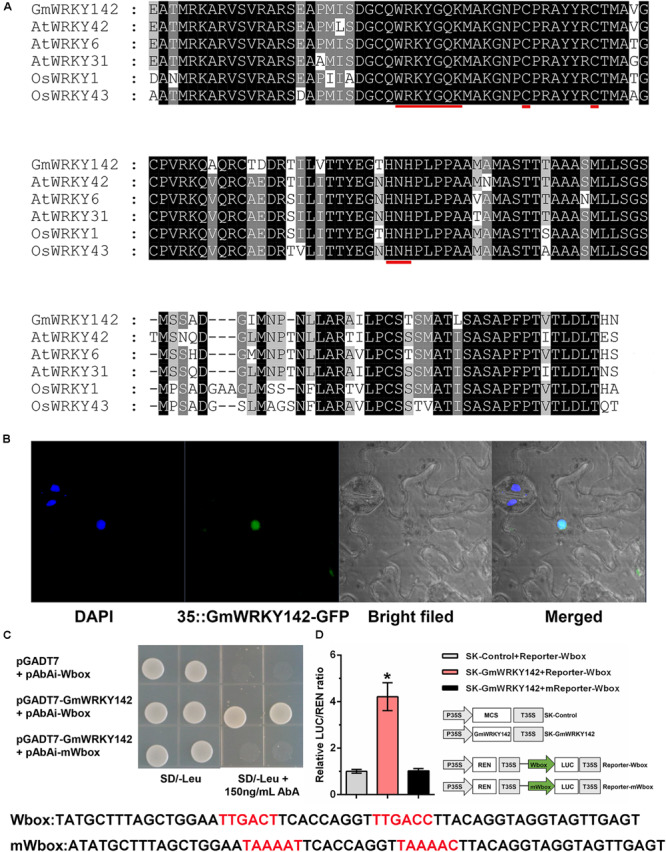
Sequence analysis, subcellular localization, and transcriptional activity assays of the GmWRKY142 protein. **(A)** Multiple alignments of the conserved WRKY domains between the WRKYs of soybean, rice and Arabidopsis. Identical amino acids are shown with a black background and analogous amino acids are shaded in gray. The WRKY motif and zinc-finger structures are denoted by red lines. **(B)** Subcellular localization of GmWRKY142 in *Nicotiana benthamiana*. DAPI was used to stain the nucleus. The overlapped images are shown on the right. **(C)** The growth phenotype of the cotransformant that harbored pGADT7-GmWRKY142 and bait vectors on a selective DO medium plate (SD/-Leu) with or without 150 ng mL^–1^ Aureobasidin A (AbA). **(D)** Schematic diagrams of the effector and reporter constructs used for transient luciferase (LUC) assays. Full-length CDS of GmWRKY142 was inserted into pGreen II 62-SK to produce an effector, while the original or mutated W-box elements were inserted into pGreen II 0800-LUC to generate reporters. MCS, multiple cloning site. P35S and T35S, the promoter and terminator of CaMV 35S, respectively. REN (Renilla luciferase) was used as an internal control for activity normalization. Transient expression assay of the promoter activity, shown as LUC/REN ratio, using tobacco leaves co-transformed with the effector and the reporters. LUC/REN ratio of the control co-transformed with the reporters and the empty effector vector (pGreen II 62-SK) was set as 1. Values are expressed as the means ± SD (*n* = 3). Significant differences according to the one-way analysis of variance are denoted as follows: *p < 0.01.

The *GmWRKY142* open reading frame (ORF) was isolated from the soybean variety Huaxia 7, based on putative sequence information available from the Phytozome database. The complete *GmWRKY142* ORF (Accession number: MN639600) spanned 1,677 bp and encoded a 558 amino acid protein with an estimated molecular mass of 59.851 kDa and an isoelectric point of 6.81. Protein sequence alignment showed that GmWRKY142 shared homology with the corresponding genes of AtWRKY6 (51.7%), AtWRKY31 (51.0%), AtWRKY42 (55.8%), OsWRKY1 (48.8%), and OsWRKY43 (44.5%). In addition, GmWRKY142 and its homologous proteins contain a typical WRKY domain that includes the highly conserved amino acid sequence “WRKYGQK,” as well as a C_2_H_2_ zinc-finger motif (Figure 2A), indicating that GmWRKY142 is a member of the WRKY transcription factor family. Further analyses were performed to confirm the status of GmWRKY142 as a typical transcription factor. The subcellular localization of GmWRKY142 was investigated, whereby the full-length *GmWRKY142* ORF without the stop codon was fused in-frame to the 5′ end of the green fluorescent protein (GFP), under the control of the cauliflower mosaic virus (CaMV) 35S promoter. The GmWRKY142-GFP fusion protein was exclusively observed in the nucleus using confocal microscopy (Figure 2B). WRKY transcription factors are characterized by their common binding activity to the cis-element W-box (TTGACC/T) in promoters of downstream genes. A tandem DNA fragment consisting of two W-box motifs (TTGACT and TTGACC) was synthesized and used to determine the transcriptional activity of GmWRKY142. In the yeast one-hybrid (Y1H) assay, cotransformants carrying pGADT7-GmWRKY142 and the pAbAi-Wbox vectors could grow on SD/-Leu plates containing 150 ng/mL AbA. However, when the W-box sequences were mutated to TAAAAT or TAAAAT, the yeast cells could not grow, which is similar to the results of the blank vector (Figure 2C). Furthermore, a dual luciferase (LUC) assay involving *N. benthamiana* leaves was performed using the constructs shown in Figure 2D. Finally, the co-expression of Effecter-GmWRKY142 with Reporter-Wbox led to a higher LUC/REN ratio than that observed in the control and mutated reporter (Reporter-mWbox), indicating that GmWRKY142 may transcriptionally activate downstream target genes.

### Overexpression of *GmWRKY142* in Transgenic *A. thaliana* and Soybean Hair Roots Confers Cd Tolerance

**FIGURE 3:**
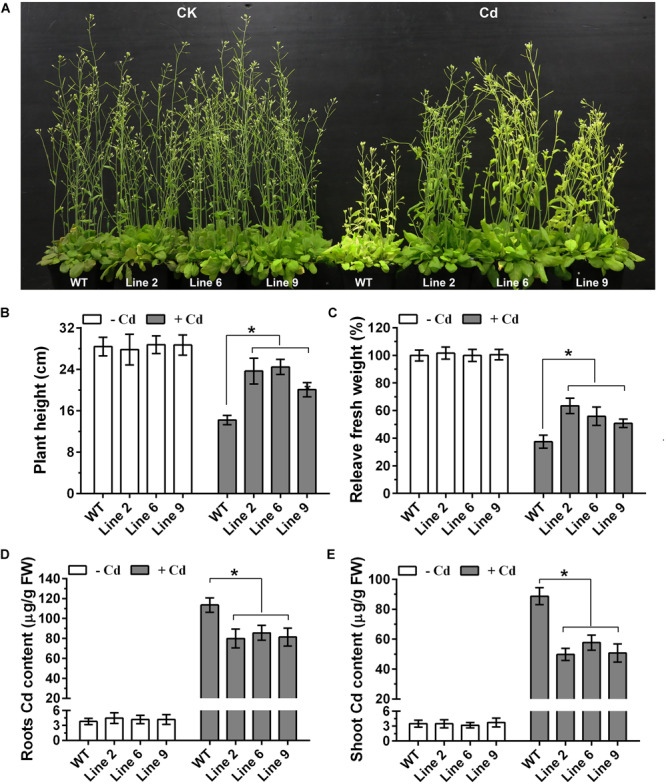
Overexpression of GmWRKY142 conferred enhanced Cd tolerance in transgenic Arabidopsis thaliana. **(A)** Phenotypes of *A. thaliana* transgenic lines (2, 6, and 9) and wild type (WT) under normal conditions or under Cd treatment conditions. **(B–E)** Plant height **(B)**, relative fresh weight **(C)**, root **(D)**, and shoot **(E)** Cd content of the WT and transgenic lines. The experiment was performed with three independent biological replicates. Significant differences according to the one-way analysis of variance are denoted as follows: *p < 0.01.

In order to determine the role of GmWRKY142 in Cd resistance, ectopic expression of *GmWRKY142* was carried out in Arabidopsis. The seeds of three independent homozygous T3 transgenic lines exhibiting a higher gene expression, as well as WT plants were collected for functional gene analyses (Supplementary Figure S1). No differences in growth and development were observed between WT and GmWRKY142-OE plants under normal growth conditions. However, under Cd stress, GmWRKY142-OE plants displayed enhanced Cd tolerance compared with WT plants (Figure 3A). Leaf chlorosis of leaves and growth inhibition are two typical symptoms of Cd stress in plants (DalCorso et al., 2008). As shown in Figure 3A, the degree of chlorosis in leaves at 10 days of exposure to Cd stress was higher in WT than in GmWRKY142-OE plants. Further evaluations revealed that the plant height and fresh weights of the GmWRKY142-OE plants were significantly higher than those of WT (Figures 3B,C), and that Cd content was higher in WT compared to GmWRKY142-OE plants (Figures 3D,E).

**FIGURE 4:**
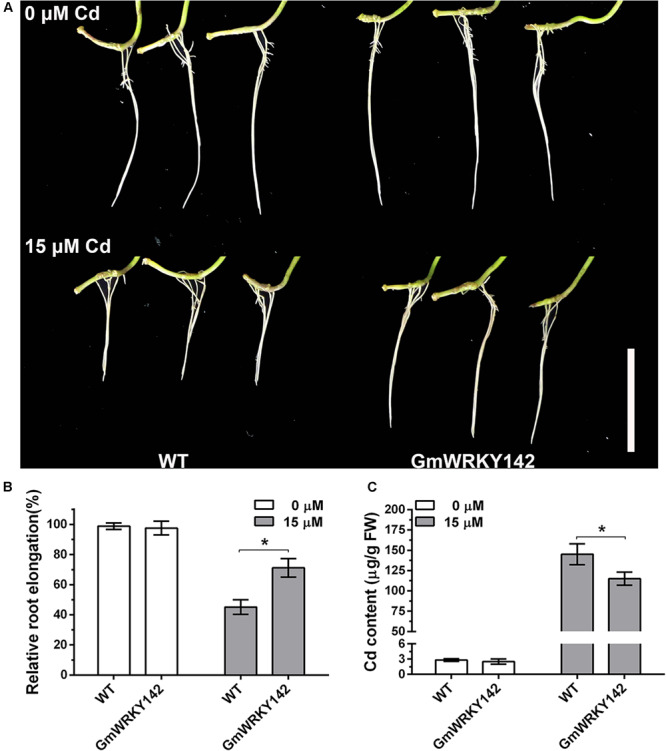
Overexpression of GmWRKY142 conferred enhanced Cd tolerance in transgenic soybean hairy roots. **(A)** Phenotypes of GmWRKY142 transgenic lines and wild type (WT) under normal conditions or under Cd treatment conditions. Relative root elongation **(B)** and Cd content **(C)** of the WT and transgenic lines were measured after 15 μM Cd treatment for 5 days. The experiment was performed with three independent biological replicates. Significant differences according to the one-way analysis of variance are denoted as follows: *p < 0.01.

To assess the effect of *GmWRKY142* overexpression on Cd tolerance in soybean, transgenic hairy roots were produced using the *A. rhizogenes*-mediated transformation system. Analysis revealed that the transcripts for GmWRKY142 were up-regulated in GmWRKY142-OX hairy roots at an average fold of 5.63 (Supplementary Figure S2). WT and transgenic soybean hairy roots, each 2 cm long, were subsequently treated with 0 or 15 μM Cd for 5 days. As shown in Figure 4, no apparent difference was found between the GmWRKY142 transgenic and WT hairy roots in the absence of Cd. However, obvious differences were observed between transgenic and control hairy roots treated with 15 μM Cd. Relative root elongation was 45.18% in the WT, compared to 75.15% in transgenic GmWRKY142 hairy roots (Figure 4B). Moreover, Cd content in the transgenic GmWRKY142 hairy roots was significantly lower than in WT roots after Cd treatment, consistent with the phenotypes of the transgenic *A. thaliana* assay (Figure 3). These combined results suggest that *GmWRKY142* overexpression in Arabidopsis and soybean hairy roots can enhance Cd tolerance.

### *GmWRKY142* Overexpression Leads to Extensive Transcriptional Reprogramming of Stress-Responsive Genes

**FIGURE 5:**
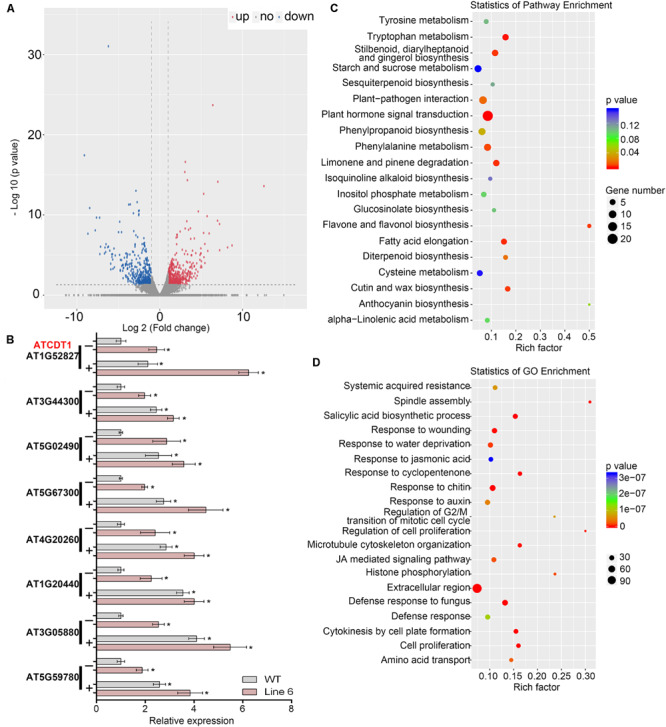
Overexpression of GmWRKY142 caused global transcriptional reprogramming in transgenic Arabidopsis thaliana. **(A)** Scatterplots of gene expression patterns in the transgenic line compared with the WT. Red represents up-regulated genes, while blue represents down-regulated ones. **(B)** Validation of the expression of eight selected Cd tolerance related genes by qRT-PCR. **(C)** KEGG pathways enriched among differentially expressed genes (DEGs). **(D)** GO analysis of DEGs. Three independent biological replicates were tested in qRT-PCR assay. Significant differences according to the one-way analysis of variance are denoted as follows: *p < 0.01.

RNA-seq was performed on an OX line (Line 6) and WT plants to determine how Cd resistance in Arabidopsis was mediated by *GmWRKY142* and also to identify potential target genes that it may regulate. The difference in expression levels between two samples was determined as log2 Fold change >1.0 and false discovery rate <0.05. A total of 746 genes showed altered transcript levels (385 up-regulated and 239 down-regulated) in the transgenic line, compared with the WT (Figure 5A and Supplementary Table S4). Data obtained from RNA-seq were confirmed by analysis, using qRT-PCR, of the transcription of eight upregulated genes which were classified into either detoxification of Cd ion (GO:0071585) or response to Cd ion (GO:0046686) genes. As shown in Figure 5B, qRT-PCR analyses on expression patterns for all of the tested genes were highly consistent with the RNA-Seq, suggesting that DEG screening based on RNA-seq is reliable.

Analysis through the Kyoto Encyclopedia of Genes and Genomes (KEGG) suggested that 68 pathways were identified in DEGs (Supplementary Table S5). The top 11 most enriched pathways with *p* < 0.05 were plant hormone signal transduction, tryptophan metabolism, fatty acid elongation, limonene and pinene degradation, stilbenoid, diarylheptanoid and gingerol biosynthesis, flavone and flavonol biosynthesis, cutin, suberin, and wax biosynthesis, phenylalanine metabolism, plant-pathogen interaction, diterpenoid biosynthesis, and, finally, phenylpropanoid biosynthesis (Figure 5C). Gene ontology (GO) analysis showed that 237 GO were significantly enriched in the DEGs (Supplementary Table S6) with the top 20 most enriched GO shown in Figure 5D. Further analyses identified 255 up-regulated DEGs containing W-box in their promoters (Supplementary Table S7), which may be direct downstream genes. We noticed that a group of downstream genes that have been shown to play direct roles in stress tolerance were up-regulated in the transgenic line, including mental stress induced proteins, auxin-responsive proteins, cell wall structural proteins and an array of transcription factors that are known as positive regulators of stress response. It should be noted that 13 genes belonging to GO:0071585 (detoxification of Cd ion) and GO:0046686 (response to Cadmium ion) were identified, with *AT1G52827* (Cadmium Tolerance 1, ATCDT1) being the only gene belonging to GO:0071585 (Supplementary Table S8). By homology search in the PANTHER (Protein Analysis Through Evolutionary Relationships) classification system^6^ using the PTHR35470 (PANTHER ID) of plant Cadmium Tolerance 1, two homologous genes in the soybean genome were identified on chromosomes 13 (*Glyma.13G361300*) and 15 (*Glyma.15G012600*), hereafter denoted as *GmCDT1-1* and *GmCDT1-2*. Compared with expression levels in control hairy root (CK), the transcription levels of both GmCDT1-1 and GmCDT1-2 were significantly increased in the *GmWRKY142*-overexpressed hairy roots (Supplementary Figure S3). These results suggest that *GmWKY142* activates *ATCDT1*, *GmCDT1-1*, and *GmCDT1-2* expression in *A. thaliana* or soybean. Further work investigated whether *ATCDT1* and its homologous genes *GmCDT1-1* and *GmCDT1-2* were direct target genes of *GmWRKY142*.

### GmWRKY142 Directly Binds to and Activates the Promoter of *ATCDT1*, *GmCDT1*-*1* and *GmCDT1-2*

**FIGURE 6:**
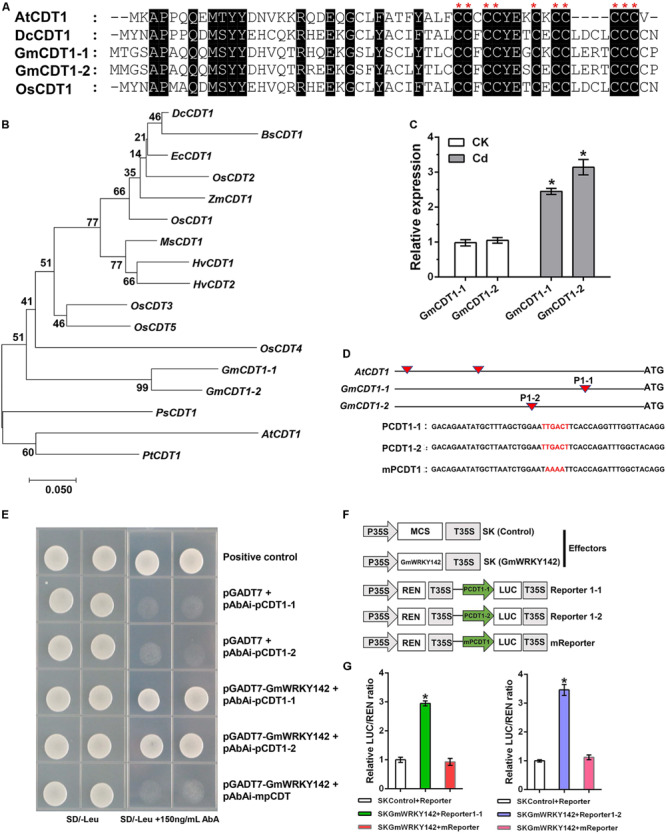
GmWRKY142 was bound to and activated the promoters of the GmCDT1-like genes. **(A)** Multiple alignments of the conserved CDT domains. Identical amino acids are shown with a black background. **(B)** Phylogenetic relationships between GmCDT1s and related proteins. Accession numbers are: AtCDT1, NM_202281; BsCDT1, ABL85053; EcCDT1, AB426477; HvCDT1, AK251605; HvCDT2, AK249080; MsCDT1, AB426478; OsCDT1, AK121052; OsCDT2, AK061597; OsCDT3, AK062450; OsCDT4, AK059641; OsCDT5, AK099514; PsCDT1, EF081525; PtCDT1, CU225257; ZmCDT1, AY103859. **(C)** GmCDT1-1 and GmCDT1-2 were induced by Cd. The Huaxia 7 roots cultured in 0 or 25 μM of CdCl2 for 6 h were sampled and qRT-PCR assay was performed. **(D)** Schematic diagrams of ATCDT1, GmCDT1-1, and GmCDT1-2 promoters and partial sequences containing W-box used in yeast one-hybrid assays. **(E)** Culture of yeast cells co-transformed with the prey and bait, as well as the positive control (pGADT7-Rec-p53+p53-AbAi) on selective medium with or without 150 ng mL^–1^ Aureobasidin A (AbA). **(F)** Schematic diagrams of the effector and reporter constructs used for transient luciferase (LUC) assays. Full-length CDS of GmWRKY142 was inserted into pGreen II 62-SK to produce an effector, while the original or mutated W-box elements were inserted into pGreen II 0800-LUC to generate reporters. MCS, multiple cloning site. P35S and T35S, the promoter and terminator of CaMV 35S, respectively. REN (Renilla luciferase) was used as an internal control for activity normalization. **(G)** Transient expression assay of the promoter activity, shown as LUC/REN ratio, using tobacco leaves co-transformed with the effector and the reporters. LUC/REN ratio of the control co-transformed with the reporters and the empty effector vector (pGreen II 62-SK) was set as 1. Values are expressed as the means ± SD (*n* = 3). The experiment was performed with at least three independent biological replicates. Significant differences according to the one-way analysis of variance are denoted as follows: *p < 0.01.

Previous studies have demonstrated that *ATCDT1* and its homologous genes (*DcCDT1* and *OsCDT1*) confer Cd tolerance to yeast or *A. thaliana* (Song et al., 2004; Kuramata et al., 2009; Matsuda et al., 2009). The amino acid sequences of GmCDT1-1 and GmCDT1-2 peptides share a high level of identity with ATCDT1, DcCDT1, and OsCDT1, and all contain 10 conserved Cys residues clustered in their carboxy-distal regions (Figure 6A). Protein sequence alignment showed that GmCDT1-1 and GmCDT1-2 shared a high amino acid sequence similarity of up to 94.6%. Two soybean CDT1 genes shared homology with the corresponding genes of AtCDT1 (58%), DcCDT1 (64%), and OsCDT1 (67%). A phylogenetic tree constructed based on the GmCDT1-1 and GmCDT1-2 and using a total of 17 CDT1 genes from different plant species shows that they could be classified into two major groups (Figure 6B). From phylogenetic analysis, GmCDT1-1 and GmCDT1-2 are found to be closest to DcCDT1, BsCDT1, ZmCDT1, OsCDTs, HvCDTs, and MsCDT1, whereas AtCDT1 forms another clade with PsCDT1 and PtCDT1 (Figure 6B). In addition, *GmCDT1-1* and *GmCDT1-2* expression levels were induced by Cd (Figure 6C), suggesting that these two genes may play a role in Cd tolerance.

Most WRKY transcription factors regulate their target stress-related genes via binding to the W-box of promoters (Ülker and Somssich, 2004; Jiang et al., 2014; Sheng et al., 2019). Isolation of the promoter sequence spanning 2,000 bp upstream of the first ATG of *ATCDT1*, *GmCDT1-1*, and *GmCDT1-2* identified 2, 1 and 1 W-boxes, respectively (Figure 6D). To test whether *GmWRKY142* can directly bind to the identified W-box, yeast one-hybrid (Y1H) was performed using *GmWRKY142* as prey, and promoter sequence fragments containing either original or mutated W-box elements as baits (Figure 6C and Supplementary Figure S4). Results showed that *GmWRKY142* could interact with selected promoter sequence fragments that contained the original W-box. However, yeast cells co-transformed with the prey and mutated W-box bait or pGADT7 and original W-box elements did not exhibit normal growth (Figure 6E and Supplementary Figure S4), suggesting the ability of *GmWRKY142* to bind to the W-box element in the *ATCDT1*, *GmCDT1-1* and *GmCDT1-2* promoters. To determine whether the GmWRKY142 transcription factor could activate GmCDT1-1 and GmCDT1-2 transcription, a dual luciferase (LUC) assay was performed in *N. benthamiana* leaves (Figure 6F). As shown in Figures 6G, the co-expression of Effecter-GmWRKY142 with Reporter1-1 and Reporter1-2 led to a higher LUC/REN ratio than that observed in the control and mutated reporter (mReporter), indicating that GmWRKY142 is a transcriptional activator of *GmCDT1-1* and *GmCDT1-2*.

### Overexpression of *GmCDT1-1* and *GmCDT1-2* in Transgenic *A. thaliana* Confer Cd Tolerance

**FIGURE 7:**
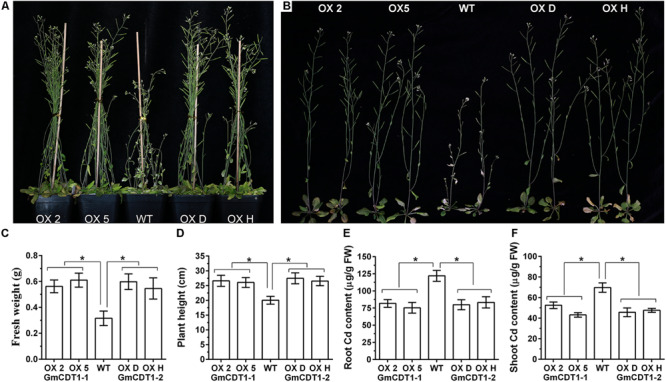
The overexpression *Arabidopsis thaliana* lines of GmCDT1-1 or GmCDT1-2 showed tolerance to Cd stress. **(A,B)** Phenotypes of A. thaliana transgenic lines and wild type (WT) after Cd treatment. **(C–F)** Fresh weight **(C)**, plant height **(D)**, root **(E)**, and shoot **(F)** Cd content of the WT and transgenic lines. The experiment was performed with at least three independent biological replicates. Significant differences according to the one-way analysis of variance are denoted as follows: *p < 0.01.

The results described above strongly support that *GmCDT1-1* and *GmCDT1-2* are direct targets of *GmWRKY142* and that they play a functional role in Cd tolerance. To explore the function of *GmCDT1-1* and *GmCDT1-2* during Cd stress, transgenic Arabidopsis and WT plants were subjected to treatment with CdCl_2_. The results showed that both *GmCDT1-1* OX lines (OX 2 and 5) and *GmCDT1-2* OX lines (OX D and H) exhibited a higher biomass accumulation than the WT (Figures 7A,B). The average fresh weight and plant height of OX lines were higher than those of the WT (Figures 7C,D). Analysis of the Cd content in the WT and OX plants under Cd stress revealed that higher Cd levels were detected in the root and shoot of WT plants compared to OX plants (Figures 7E,F). These results suggest that *GmCDT1-1* and *GmCDT1-2* overexpression in Arabidopsis can enhance Cd tolerance by limiting Cd uptake.

## Discussion

Heavy metal stresses such as high concentration of Cd, Cu and As are among the major environmental factors which not only limit the productivity and growth potential of plants, but also cause serious problems for human health (Gallego et al., 1996; der Maur et al., 1999; Matsuda et al., 2009). Fortunately, a number of strategies have been employed in plants to resist heavy metal stress, including uptake, accumulation, translocation, and detoxification of the metal. Various transcription factor-mediated metal stress sensing and coping strategies have been reported in plants (Liu et al., 2017; Zhu et al., 2018). The WRKY transcription factor family is one of the most important regulator families reported to be involved in a variety of heavy metal stresses. For example, several studies have revealed the differential transcript expression of WRKY transcription factors under Cd stress in different plant species (Yang et al., 2016; Hong et al., 2017; Cheng et al., 2018; Han et al., 2019; Sheng et al., 2019). An increasing number of WRKYs that were identified to confer Cd tolerance in plants also exemplify the importance of heavy metal tolerance in plants. To date, no report on the role of soybean WRKYs in Cd detoxification and tolerance has been published. In the present study, 29 GmWRKYs were detected as Cd-responsive DEGs using genome wide transcriptomic analysis in roots (Table 1). Of these, 26 GmWRKYs were up-regulated and 3 were down-regulated, suggesting that functional differentiation may exist. We further demonstrated that *GmWRKY142* is involved in Cd resistance and provided several lines of evidence supporting this observation. Firstly, GmWRKY142 is a nucleic protein with transcriptional activation activity and its expression was rapidly induced by Cd in a dose-dependent manner (Figures 1, 2). Secondly, *GmWRKY142* overexpression in Arabidopsis and soybean hair roots resulted in improved Cd resistance, indicating that *GmWRKY142* is a positive regulator of Cd tolerance (Figures 3, 4). Thirdly, *GmWRKY142* overexpression lead to the extensive transcriptional reprogramming of stress-responsive genes. We noticed that several downstream genes that have been shown to play direct roles in stress tolerance were prominently influenced by *GmWRKY142* overexpression including crucial metabolic regulators, mental stress induced proteins, auxin-responsive proteins, cell wall structural proteins and an array of transcription factors. One of them, the salicylic acid biosynthetic

process (GO:0009697), which included 22 up-regulated and 9 down-regulated genes, had previously been found to be significantly associated with Cd tolerance in plants (Belkhadi et al., 2010; Chao et al., 2010; Szalai et al., 2013), and was enriched in the transgenic line (Figure 4 and Supplementary Table S9). Finally, the present study demonstrated that the GmWRKY142-AT/GmCDT1 cascade module confers Cd tolerance (Figures 5, 6). These findings suggest that GmWRKYs act as novel participants in the response of soybean to Cd stress, although further studies will be required to support this conclusion.

WRKYs can activate or repress the transcription of stress-related and co-regulated genes by directly recognizing and binding to the W-box element within the promoters of target genes (Eulgem et al., 2000; Ülker and Somssich, 2004; Han et al., 2019). In the present study, we demonstrated that overexpression of *GmWRKY142* in transgenic *A. thaliana* or soybean hairy roots lead to enhanced tolerance to Cd and activated the expression of the *ATCDT1* or *GmCDT1*-like genes (*GmCDT1-1* and *GmCDT1-2*) encoding Cd-binding Cys-rich proteins (Figures 3, 5 and Supplementary Figure S3). The *ATCDT1*, *GmCDT1-1*, and *GmCDT1-2* promoters were found to contain 2, 1, and 1 W-box elements, respectively (Figure 6C). We further demonstrated that *GmWRKY142* activated the *ATCDT1*, *GmCDT1-1*, and *GmCDT1-2* promoters through interactions with the W-box element using the yeast one-hybrid or dual luciferase (LUC) assays (Figures 6D,E and Supplementary Figure S4). So far, a plethora of target genes have been characterized for various WRKY members. However, only a limited number of WRKY TFs and their target genes were identified to regulate Cd tolerance in plants. *AtWRKY13* is induced by Cd stress and directly activates the expression of the Cd^2+^ extrusion pump gene *AtPDR8*; therefore, *AtWRKY13* overexpression in transgenic *A. thaliana* leads to an enhanced tolerance to Cd (Sheng et al., 2019). *AtWRKY12* as a negative regulator directly targets *GSH1* and indirectly represses PC synthesis-related genes expression to negatively regulate Cd accumulation and tolerance in Arabidopsis (Han et al., 2019). These two studies show that WRKY proteins can act as transcriptional activators or repressors to regulate Cd accumulation and tolerance. Herein, the characterization of *ATCDT1* and *GmCDT1*-like genes as direct targets of *GmWRKY142* provides valuable clues to better understand the WRKY regulatory mechanisms associated with Cd stress. However, RNA-seq results indicated that *ATCDT1* and *GmCDT1*-like genes were not the only identified target genes (Figure 5), thereby highlighting the importance of confirming the identity of the other target genes to better understand the molecular mechanism of *GmWRKY142* in Cd tolerance.

In the previous studies, three members of the CDT1 family, namely, DcCDT1, OsCDT1 and ATCDT1, from *Digitaria ciliaris*, *Oryza sativa* and *A. thaliana* were isolated and characterized for Cd tolerance (Song et al., 2004; Kuramata et al., 2009; Matsuda et al., 2009). In contrast to other Cys-rich peptides, CDT1 peptides are only present in angiosperms and gymnosperms, and are relatively small in size with 49 to 60 amino acid residues (Song et al., 2004). In the present study, two homologous CDT1s were identified in soybean and their expression induced by Cd stress (Figure 6B). Consistent with *DcCDT1*, *OsCDT1*, and *ATCDT1*, overexpression of *GmCDT1-1* or *GmCDT1-2* conferred Cd-tolerance to transgenic *A. thaliana* plants by lowering Cd accumulation in plants (Figure 7). A conserved sequence with 10 Cys-rich peptides, CC-(C/F)-CCYE-X-C-(K/E)-CC-(LDCL/LERT/delete)-CCC-(V/C), in their C-termini was identified in this study. Plants have evolved a set of versatile adaptive mechanisms to cope with Cd stress. One such means is by reducing the cellular accumulation of Cd by either enhancing Cd efflux or suppressing its uptake. The Cys-rich membrane proteins in different plant species have been found to play an important role in plant Cd resistance by chelating Cd at the cellular surface and limiting further entry of Cd into the cells (Song et al., 2004; Kuramata et al., 2009; Matsuda et al., 2009; Pirzadeh and Shahpiri, 2016; Niederwanger et al., 2017). Here, we have demonstrated that expression of GmCDT1-1 or GmCDT1-2 conferred Cd tolerance to Arabidopsis plants by reducing their cellular Cd contents. GmCDT1-1 and GmCDT1-2 are Cys-rich peptides, so it is highly likely that they can directly bind Cd. Based on our current findings, therefore, the most plausible mechanism we envisage for GmCDT1s and AtCDT1 function is that these proteins chelate Cd at the cellular surface and prevent further entry of Cd into the cells. These findings suggest that CDT1s play an important role in Cd tolerance.

**FIGURE 8:**
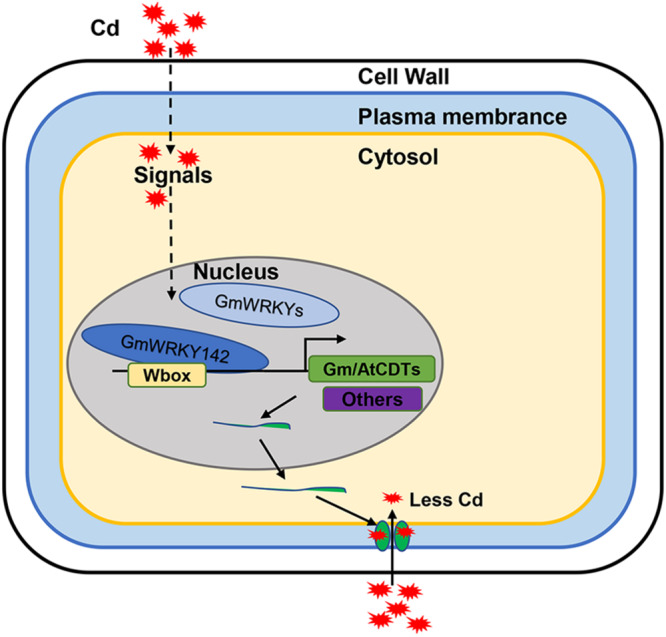
A working model for the role of GmWRKY142 in regulating Cd tolerance. Cd stress promotes GmWRKY142 expression and thereby directly activates the transcript of the GmCDT1-1 and GmCDT1-2 genes encoding Cys-rich peptides, which results in chelating Cd at the cellular surface and prevent further entry of Cd into the cells, and thus, conferring enhanced tolerance.

In summary, 29 Cd-responsive WRKY genes were identified and confirmed in soybean roots, of which 26 were up-regulated and 3 were down-regulated. Among these GmWRKYs, the up-regulated *GmWRKY142*, in particular, directly activated expression of the *ATCDT1* and *GmCDT1*-like genes, which resulted in reduced Cd accumulation, thereby conferring Cd tolerance (Figure 8). The identification of 29 Cd-responsive GmWRKYs as well as the GmWRKY142-CDT1 cascade module provides the first step in determining that GmWRKYs may be critical factors in Cd tolerance. More GmWRKYs-targeted cascade modules conferring Cd tolerance need to be further studied.

## Data Availability Statement

The data sets supporting the results of this study are included in the article. The RNA-seq data have been deposited into the NCBI Short Read Archive (SRA, https://www.ncbi.nlm.nih.gov/sra/) under accession number PRJNA605520.

## Author ContribUtions

ZC, PX, HW, and RL performed the experiments and data analyses. ZC and HN prepared the manuscript. HN planned, supervised, and financed this work. YC, TL, and QM reviewed and edited the manuscript. All authors have read and approved the final version of the manuscript to be published.

## Conflict of Interest

The authors declare that the research was conducted in the absence of any commercial or financial relationships that could be construed as a potential conflict of interest.

## Footnotes

1 ftp://ftp.ncbi.nlm.nih.gov/genomes/all/GCF/000/004/515/GCF_000004515.5_Glycine_max_v2.1

2 ftp://ftp.arabidopsis.org/home/tair/Sequences/whole_chromosomes/

3 http://www.geneontology.org

4 http://www.kegg.jp/kegg

5 https://phytozome.jgi.doe.gov/pz/portal.html#

6 http://www.pantherdb.org/
