# Supplementary material for: Transcription Factor GmWRKY142 Confers Cadmium Resistance by Up-Regulating the Cadmium Tolerance 1-Like Genes
Source: Front Plant Sci. 2020 Jun 3;11:724. doi: 10.3389/fpls.2020.00724 (PMC7283499; doi:10.3389/fpls.2020.00724)
Supplement: Supplementary file 2 [file Data_Sheet_2.PDF]

Supplementary Material

1 Supplementary Figures and Tables

1.1 Supplementary Figures

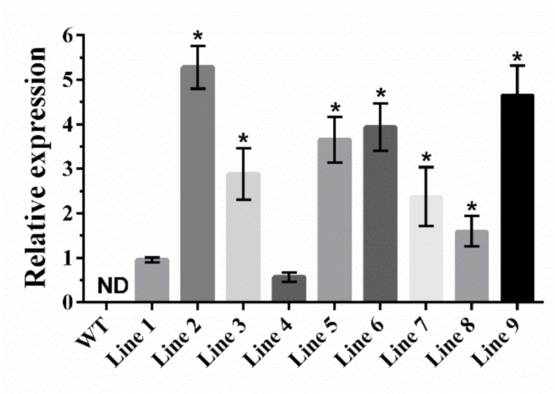

Supplementary Figure S1. Quantitative real-time PCR analysis of overexpressing *GmWRKY142* Arabidopsis lines. ND, not detected. Data are means ± SD of three biological replicates. The * indicate statistically significant difference, using one-way ANOVA and Duncan's test (*P*≤0.05).

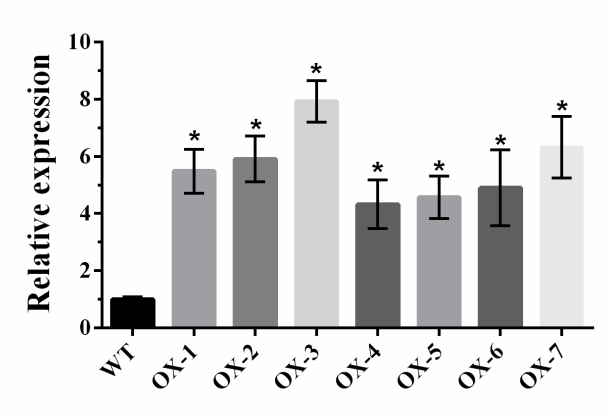

Supplementary Figure S2. Quantitative real-time PCR analysis of overexpressing *GmWRKY142* soybean hairy roots. Data are means ± SD of three biological replicates. The * indicate statistically significant difference, using one-way ANOVA and Duncan's test (*P*≤0.05).

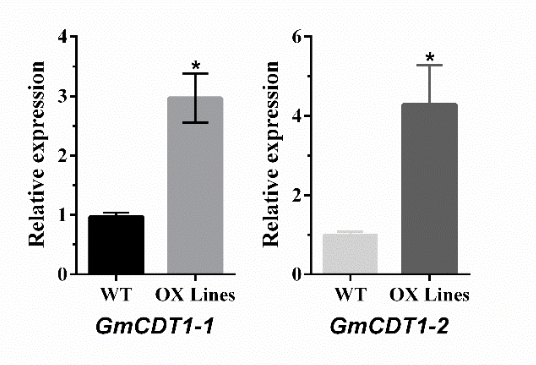

Supplementary Figure S3. The expression of GmCDT1-1 and GmCDT1-2 in overexpressing *GmWRKY142* soybean hairy roots. Data are means ± SD of three biological replicates. The * indicate statistically significant difference, using one-way ANOVA and Duncan's test ($P \leq 0.05$).

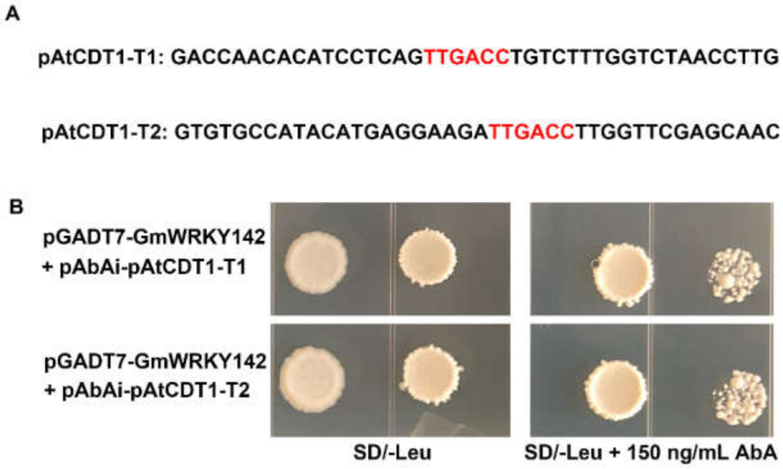

Supplementary Figure S4. GmWRKY142 was bound to and activated the promoters of the ATCDT1. (A) Schematic diagrams of ATCDT1 promoters and partial sequences containing W-box used in yeast one-hybrid assays. (B) Culture of yeast cells co-transformed with the prey and bait on selective medium with or without 150 ng mL-1 Aureobasidin A (AbA).

2 Supplementary Data

2.1 CDS sequencing information of *GmWRKY142*

ATGGACAAAGGATGGGGACTCACCCTTGATACCTCTTCCTCTCAATCTCTTCCATTATTC
CCTTCCAACGATAATAAGATGTTTCCTCTCCTCGGATTCCCCGTCAACCTCAGCCGCGCC
TCCAAGGAAGACGATGAAAACCGCAAAGTTGTCGGTGAAGTTGACTTCTTCTCTGATAG
AAACAAACCTACACCTCCACCCTCTCACGACCACAACGTTAAACCCAACATCGTCAAGA
AGGAAATCGATGAAACACCTCTTCACATTAATACTGGTTTACAACTCCTTACTGCTAACA
CCGGGAGTGACCAATCTACCGTGGATGACGGGGTTTCATCTGATGCAGAAAATAAGCGA
GCCAAAACCACTGAGCTTGCACAGTTGCAGGTGGAGCTTCAACGCATGAACTCTGAGAA
CAAGAAGCTGAAAGAGATGCTCAGCCATGTGACCGGTAACTACACCGCCTTGCAGATGC
ATCTTGTCACATTAATGCAACAGAACCAGCAGCGAACAGGAAGCACGGAAAATGAGGT
TGTTCAGGGAAAGGTAGAGGATAAAAACGTTGGTGTTGGTGGAGGGAAGGTACCAAGA
CAGTTCCTTGATATAGGTCCCAGTGGCACAGCAGAAGTAGATGATCAAGTTTCGGATTC
TTCTTCTGATGAAAGAACACGATCGAGCACACCTCAGAACCATAACATTGAAGCTGGAG
CTAGAGATGGTGCGAGAAATAACAATGGTAAAAGCCAGTTGGGTAGGGAAGAGAGTCC
AGACTCAGAATCACAAGGTTGGGGTCCCAATAAGCTTCAGAAAATGAACCCTTCCAACC
CTATGGATCAATCCACTGCAGAAGCCACAATGAGAAAAGCTCGTGTATCAGTTCGTGCC
CGATCAGAAGCTCCCATGATCAGTGATGGATGCCAATGGAGAAAATATGGACAAAAAA
TGGCCAAGGGGAATCCATGCCCTCGAGCATACTACAGATGCACTATGGCAGTTGGTTGC
CCAGTTCGCAAACAAGTTCAACGTTGTGCTGATGATAGAACCATTTTGGTTACAACATAT
GAAGGCACGCATAACCATCCACTGCCACCTGCTGCTATGGCCATGGCATCAACCACTAC
AGCTGCTGCTAGCATGTTGCTTTCTGGATCAATGTCCAGTGCAGATGGAATAATGAACCC
AAATTTGCTAGCTAGAGCAATTCTTCCTTGCTCTACAAGCATGGCAACACTCTCAGCTTC
AGCACCATTCCCCACTGTGACTTTGGACCTCACACACAACCCTAACCCATTGCAATTTCA
AAGGCCTGGTGCCCCATTCCAAGTACCCTTCCTTCAAGCACAGCCTCAGAACTTTGGGTC
AGGAGCCGCCCCAATTGCACAAGCACAAGCACTTTATAATCAATCAAAATTCTCTGGTC
TTCAGTTGTCTCAGGATGTAGGATCTTCTCAATTAGCACCACAAGCTCCTAGACCACCCT
TACAGCCAAGCCAACAGCCCTCACTTGCTGACACAGTCAGCGCCGCCGCCTCCGCCATC
ACCGCCGATCCAAACTTCACCGCCGTGCTTGCCGCCGCCATCTCCTCCATCATAGGCAGT
GCTCATAATTCAAACAACAACAACAACAACAACAACAACACAAGCAGGACTACTATTA
GCAGCTTTTCAGGAAACTGA

2.2 CDS sequencing information of *GmCDT1-1* and *GmCDT1-2*

> GmCDT1-1
ATGACGGGTTCTGCACCAGCACAGCAGCAGATGTCATACTATGATCACGTTCAGACGCG
CCATCAGGAGAAGGGAAGCCTCTATTCTTGTTTGTATACACTCTGTTGCTGCTTTTGCTG
CTATGAGGGTTGCAAGTGCTGCTTGGAACGCACCTGTTGTTGCTGTCCCTAG

> GmCDT1-2
ATGACGGGTTCTGCACCAGCACAGCAGCAGATGTCATACTATGATCACGTTCAGACGCG
CCATCAGGAGAAGGGAAGCCTCTATTCTTGTTTGTATACACTCTGTTGCTGCTTTTGCTG
CTATGAGGGTTGCAAGTGCTGCTTGGAACGCACCTGTTGTTGCTGTCCCTAG

2.3 Promoter sequencing information of *GmCDT1-1* and *GmCDT1-2*

> The promoter of GmCDT1-1

GTTATATCATGAGTATGCATGTAATGTATGACTTGGCTTTGTGCTAGCTAGTTATTCTAC
CCCCAGTTTTCTAGTTGTTAGATAAATTCACCTGATCATTTTATGTTTCATTTCATAATTC
ATAATATTTCTATACTAGCTCGCTCCTGAATTTTTATAGGTTAATATAACATCAGGATAT
ACTTTAAATCTATTCTTATTCTTCACTTTTAATGAGAAATGGATTTATCCTTAGTTGCTAA
TAATTTTAGAGACAAACTCATTTTTAATCAAAGAATATGGATTTATCCTTCATCAATTAT
ATGCAGCGAATATGATTTATATATAAAACAGATTTAAAAAACTATGGATCCAGCTATTT
GATTATATATTTTTATTTTTTTGAAGAATATTTATAGAATGAACAAGGAGAAAAAACTTT
GCTCGTACGAAGAATGAACAAGAGAGTAATATAGACATAGTATCACTTTAAAAAAATAC
CAATGAAAACAGTTTAATGGACTAGTTCTTCAAAATATGAAGATAAATATCGAGGGGTT
AGGGATTTGAAATATAGCTTGCATAAGGGGGTCCTAGCTTCTAACCTTATTCTAGTCATT
GTCGTTATTGTACACCAAAAAATAAGATAAATTAATAAAAAAATTGTTATTAAATGATT
TATTTTTGTAATATATTTATTAGATAGTAATTAAATAATAAAATGAATTATTCATTTTTTT
ATAGCAAAAAGAGGTTAAAGATATTATCAAAAACCTAGTGAAATATGTTCTCAGTTTTC
TATGGTAATTTTTTTAATCGATAAAAGGTTTACAAGATAATTAAAAAAGATTATATATTT
CCATGTTTTATTTTAAGCTTAAACACATTTTCAGATAGTATAAAGATAACATCTTTTTTAT
TTTGGTTCTTCGTTATTATTATTATTTATTTATTTTTGCCGCGGTCAAAGAATAAAGAGTT
TAATGTTAGTCTATGATTAACTTGTTTCTGTTAAGTAACCAGGGTTAAGTGATGATAAAA
TGCTACATCATCACTACCGGGTCTCATAACAAAAAATAAAAAATTGTAACATTTATAAG
AAGAAACAACATATTCAAACCAATTACTGTAATTAGCTGGGTTCATATATGCAACGTTC
CGCTCGCACCGGTTGGGGAGGAAGTAATGGTTGAGGTTGAGGAGATGTTTCAACATATG
ACTCTTGCCAGTATAAAATAGTTTGAGAGTTTTGAACTATCATAGTAGGGCAGAGTCAA
GCGTACTAGTGTGATTGATTTTGGAGTAAAACTAAACGGGCCCAAGTCTTATGGCCGGA
GTCTGATACTAAGAATTTCCAACACAAAATTCTATATTGGTGGACTTACTTCGGGTATAA
TGTGATTCAAACTTTACTAGCTCGATCAATTATTATTCCTAACTGTTAACGTATAAAATG
GATTTCTTTTGACAGAATATGCTTTAGCTGGAATTGACTTCACCAGGTTTGGTTACAGGT
AGGTAGTTGAGTTTCTTGCGCAATGAATGGGTTTGAACTTTGAAGGGTGGTTAGGTGTCA
GTATGAGAGTTGATAATAAATTGGTGCACGGACCAAGTATGCTTCCATATGTGTATTATT
ATGATGGATGGGTTGTTCTTTTCTATTATCCAAGTGACTCGTCGTTATTCAATTATTAAAT
TTTACAAAATTCAAATTATTTCTTATAACAAGTCGTGTGTAATACTTTCTATTTTCTATTA
ATCATGTGGTAACTAATATACTTGTCATTGCTTGAGTTATTTTGTTTAACAGAAAATGGT
ACTATATCATACAAGAAAACATTTAGAAAAAACTATTTGAGCAATTCTTGTATAGACAA
GTGATCATCAACTGTACTTAGCTAAGTGTTTGGCACCATTCTGGGGTTGGTTTCATGACT
TTTCCAGCCACTGAAGCCAACCAATTCCTTTCTACTAACTACCCTAATAAATGGCCGGCG
AGAAAGGCAAAAAGAGGGGATGATGGTGGAAGCCTTAACATGTTCAAGCAACTTTCTGT
GCTTCAGCTTATAAAATAAATGTATGGTCCAACACTGGTGCATTAATCATCTTCTTCAAA
TCCCAAAAT

The promoter of GmCDT1-2

TATACAACGTGCTTCTGTTATATTCCGCTCGCACCGGTTGTGGAGGAAGTAATGGTTGAG
GTTGAGGAGATGTTTCAACATAGGAGTATGAAATGACTCTGGGCAGTATAAAATCGTTA
AAAAGAGATTTTGCCAGTTCTTAACTATCATAATTTTTAAAAAAAATGAGGCTTTGTTTG
TGTTGTCACCTGATTGTGCTTATTTATTTAATGGTTTTACATAGTTAGTTTCAGTTTAGTA
CTGATTTTAAAAGATTAGGATGTAATTTAATTGCTTTAAGCGACTTTAAATCACTAGGAT
AAAAGGCTATATAATTATAATTATAGAGATTAAAATGAGATATTTTAAAATATGAGAAC
TAAAATTAAGATGTATAAAAAAATGATTACGTTAAGAAGAATTTGAAGCCCGGTGAATG
AATTCGATTATTTTAGGCTGAGCAATTTTTTCCCTGTATGTTTCATCCAATATGTTCCAAA
TAAAAGCCTGAAATTCAACTACTAAAGCCCAAGTTCAATCATTATCATGCATTTAAAAA
GACAACTCAATTTAATTATGTGCATTCTCTTCCCCTTCGAAAGAAATTATTCCCCGTCCTT

TCCTTTTTATCTTGCTCTATGGCACCAAGAAAACAATCTCGTGTACCTGCCCAGTATTCTC
CCTCGAGACTTCAATGCTAATTATAAATACCGAATCTATGCTTGGAAATTGGAAGTTCGA
CTCGTCTCTCTTTTAAGCCACCGCAAATGTTTGATTCCATTTGTTTTAAATTTTAATAAAT
TTGAATATAAACTATTAAATGTAATTGAAATATGTGTGTAAATCAATCTTAATAACAATT
TTTATATTATAATGTATGTCTCCTAGTGTGAAATTGATTTTGGAGTAAAACTAAACAGGC
CTAAATCTTATGGCTGGAGTCTGAAATTTAGAATTCAAGGCAATGTAAAATTTCCAACTT
ACTTCAGGTATGATGTGATTCAAACTTTACTAGCTCGTTCAATTATTATTCTTAACTGTTA
AAGTATAAAATGGATTTCTTTTGACAGAATATGCTTAATCTGGAATTGACTTCACCAGAT
TTGGCTACAGGTAGGTAGTTGAGTTTCTTGCGCAATGAATGGGTTTGAAGGGTGGTTAG
GTTGCAGTATGAGAGTTGATAATAAATTGGGGCACGGACCAGGTATGCTTCCATATGTG
TGTTATTATGAATTTATGATGGACGGGTTGTTCTTTTCAATTATCTAAGTCAGTCGTTGTT
ATACAATTATTAAGTTTTATAAATTTAAATTATTTCTTGTAACAAGTCATGTGTAAAACT
TTCTATTGTCTATTAATCATGTGGTAACTAATATGCCTGTCATTGTTTGAGTTTTATGCTT
TCTTGAGATTTTTTTTATAAGAAAATTATATATATATATCATACTAGAAAACATTTAGAT
ATGTGATCATCAACTATACTTAGCTAAGTATGCAAATTTCTTTTTTGGAAGAAATATAAG
GTTTGGGGTCAGAACTTTTTTTTTCTTAATGAAACAAACCAACTATGTCCAACACTAATA
TAATGTGGTATAGTTGGTAGATAGATTGGAGATTCATGTGTATAGAGGATTAGAAGAAT
CAGTCTCTTAAATGAATTTTACTATCTCTAGAGATTAGTTTCTAATCCTAGAACATCAGT
CTCTTAAATGAATCTTAATTAAATTAGTGACTGTGACATCTATCCACCATCAAAACACAT
TAGTAACTTTATAAGTAGAACTATGATTGATTTTTACTGAGAATTATGGTCTTTCCCACA
TTCTGGGGTTGGTTTCATGAGTTTTCCAGCATCTGAAGCCAACCAATTCCTTTCTACTAA
CTACCCTAATAAATGGCAGGCGAGAAAGGCAAAAGGAGGGGTTGGGGGTGGAAGCCTT
AACATGTTCGAGCAACTTTGTGTGCTTCAGCTTATAAATGAATGTATGGTCCAACACTGT
GCATGAATCATCTTCTTCAAATCCCAAAAT
